# Exaggerated Nighttime Sleep and Defective Sleep Homeostasis in a *Drosophila* Knock-In Model of Human Epilepsy

**DOI:** 10.1371/journal.pone.0137758

**Published:** 2015-09-11

**Authors:** Emily Petruccelli, Patrick Lansdon, Toshihiro Kitamoto

**Affiliations:** 1 Interdisciplinary Program in Genetics, University of Iowa, Iowa City, IA, United States of America; 2 Department of Anesthesia, Carver College of Medicine, University of Iowa, Iowa City, IA, United States of America; Biogen Idec, UNITED STATES

## Abstract

Despite an established link between epilepsy and sleep behavior, it remains unclear how specific epileptogenic mutations affect sleep and subsequently influence seizure susceptibility. Recently, Sun *et al*. (2012) created a fly knock-in model of human generalized epilepsy with febrile seizures plus (GEFS+), a wide-spectrum disorder characterized by fever-associated seizing in childhood and lifelong affliction. GEFS+ flies carry a disease-causing mutation in their voltage-gated sodium channel (VGSC) gene and display semidominant heat-induced seizing, likely due to reduced GABAergic inhibitory activity at high temperature. Here, we show that at room temperature the GEFS+ mutation dominantly modifies sleep, with mutants exhibiting rapid sleep onset at dusk and increased nighttime sleep as compared to controls. These characteristics of GEFS+ sleep were observed regardless of sex, mating status, and genetic background. GEFS+ mutant sleep phenotypes were more resistant to pharmacologic reduction of GABA transmission by carbamazepine (CBZ) than controls, and were mitigated by reducing GABA_A_ receptor expression specifically in wake-promoting pigment dispersing factor (PDF) neurons. These findings are consistent with increased GABAergic transmission to PDF neurons being mainly responsible for the enhanced nighttime sleep of GEFS+ mutants. Additionally, analyses under other light conditions suggested that the GEFS+ mutation led to reduced buffering of behavioral responses to light on and off stimuli, which contributed to characteristic GEFS+ sleep phenotypes. We further found that GEFS+ mutants had normal circadian rhythms in free-running dark conditions. Interestingly, the mutants lacked a homeostatic rebound following mechanical sleep deprivation, and whereas deprivation treatment increased heat-induced seizure susceptibility in control flies, it unexpectedly reduced seizure activity in GEFS+ mutants. Our study has revealed the sleep architecture of a *Drosophila* VGSC mutant that harbors a human GEFS+ mutation, and provided unique insight into the relationship between sleep and epilepsy.

## Introduction

Substantial evidence supports an intimate reciprocal relationship between sleep and seizures. On one hand, the sleep state has a significant impact on seizure activity [1, 2]. For example, seizures can be facilitated by the neuronal synchronization that typically occurs during non-rapid eye movement (NREM) sleep [3]. Also, sleep deprivation is generally considered to trigger or worsen seizures in patients with epilepsy [4, 5]. On the other hand, seizures often influence the quality and quantity of sleep, causing irregular sleep patterns or circadian disruption in epileptic patients [6, 7]. Furthermore, antiepileptic drugs commonly affect sleep [8], making the interactions between sleep and seizures even more complex. Altogether these interactions can set into motion a vicious cycle of seizures and sleep abnormalities. Despite this well-recognized interplay between seizures and sleep, the neurobiological underpinnings of this relationship remain largely elusive. A better understanding of the sleep-seizure relationship is thus expected to provide important insights into seizure pathophysiology and sleep mechanisms.

Although evolutionarily distant from mammals, the fruit fly *Drosophila melanogaster* has emerged as a powerful model to study fundamental molecular and cellular processes underlying sleep/wake behavior [9–12]. Defined as five minutes or more of inactivity, fly sleep embodies many characteristics of mammalian sleep. Specifically, the sleep state of flies is: 1) subject to circadian and homeostatic regulation, 2) associated with increased arousal thresholds and altered brain activity, 3) susceptible to sleep-wake drugs, and 4) controlled by highly conserved molecular pathways such as inhibitory GABAergic signaling [11–15]. In *Drosophila*, a number of mutants display seizure-like neuronal activities, and their behaviors have been molecularly and physiologically characterized [16, 17]. These mutants include loss-of-function forms of voltage-gated potassium channel subunit genes, *Shaker* (*Sh*) and *Hyperkinetic* (*Hk*), and their modulator *quiver*/*sleepless* (*qvr*/*sss*). Interestingly, these seizure-prone mutants have significantly reduced sleep [18–20]. In addition, Lucey *et al*. recently found that sleep deprivation increases the seizure susceptibility of fly mutants in which seizure-like activity is induced by either mechanical or temperature stress [21]. Taken together, these results indicate that, as observed in human epilepsy patients, sleep and seizure activity are functionally related in *Drosophila*.

The most common mutations associated with human epilepsy occur in the voltage-gated sodium channel (VGSC) gene *SCN1A*. VGSCs are essential for the generation and propagation of action potentials, making them integral players in defining the excitability states of neurons under both physiological and pathological conditions [22]. So far, more than 600 different *SCN1A* mutations of varying deleterious effect have been found to result in a broad spectrum of epileptic disorders, including generalized epilepsy with febrile seizures plus (GEFS+) [23, 24]. GEFS+ is typically an autosomal dominant disorder hallmarked by febrile seizures (short tonic-clonic attacks during a >38**°**C fever) that persist beyond childhood and can eventually manifest regardless of temperature. Also, human VGSC mutations and GEFS+ *Scn1a* mouse models have been linked to various sleep defects [25–28]. These findings highlight the importance of VGSC function in the relationship between sleep and epilepsy.

In *Drosophila*, VGSCs are encoded by a single gene *paralytic* (*para*), and Sun *et al*. recently created a knock-in fly model of GEFS+ by inserting the human GEFS+-causing *SCN1A* mutation (*SCN1A* ^*K1270T*^) into the corresponding fly locus (*para* ^*K1353T*^) [29]. *Drosophila* GEFS+ mutants exhibited semidominant heat-induced seizure-like behavior, likely due to reduced GABAergic inhibitory activity in the central nervous system at high temperatures (>35**°**C). Electrophysiological analysis revealed that upon temperature elevation the gain-of-function GEFS+ mutation increased sodium currents, leading to sustained depolarization of GABAergic neurons and reduced inhibitory activity. GEFS+ channels also showed reduced activation thresholds regardless of temperature, which could enhance GABAergic signaling at room temperature [29].

Here, we characterize the sleep/wake activity of a fly model of GEFS+, revealing the effects of altered VGSC activity on sleep and probing the possible interactions between sleep and seizure behavior. We report that this gain-of-function VGSC mutation leads to a significant increase in nighttime sleep and to rapid sleep onset after lights off. Both pharmacologic and genetic manipulations implicate increased GABAergic signaling in the enhanced sleep of GEFS+ flies. We also observed that the GEFS+ mutation dominantly disrupted homeostatic sleep regulation, and unexpectedly found that sleep deprivation reduced the susceptibility of GEFS+ mutants to heat-induced seizures. Overall, our study describes a unique sleep profile of a seizure-prone *Drosophila* VGSC mutant, and demonstrates the value of fly models for investigating the sleep-seizure interaction.

## Materials And Methods

### Fly Husbandry

Flies were raised under the conditions of a 12 hr light/dark cycle, at 25°C and 65% humidity on standard cornmeal agar food. For behavioral analyses, newly eclosed flies were collected under CO_2_ anesthesia over a two-day period, housed 20/vial (all virgin females or 10 female/10 males), and aged for 3–4 days prior to experimentation. GEFS+ mutant (*w para* ^*GEFS+*^; *UAS-GFP*) and control flies (*w*; *UAS-GFP*) were obtained from Dr. Diane O’Dowd (University of California, Irvine) [29]. GEFS+ mutants and their controls have comparable genetic backgrounds because homologous recombination was performed in parallel with GEFS+ (K1270T) and wild-type (K1270K) donor sequences [29]. *Canton-S* and *w* ^*1118*^ flies were collected from our common lab stock. The *pdf-GAL4* strain was shared by Dr. Bridget Lear (University of Iowa) and the *UAS-Rdl-RNAi* (v41103) line was acquired from the Vienna *Drosophila* Resource Center.

### Basal Sleep Analysis

The *Drosophila* Activity Monitor (DAM) system (TriKinetics, Waltham, MA) was used to record locomotor activity (infrared beam breaks) of individual flies in one-minute bins, and sleep was defined as any inactive bout lasting ≥5 min [12]. Basal sleep analyses were carried out using DAM2 monitors in a Fisher Scientific incubator (56 x 61 x 71 cm) at 25°C and ~40% humidity. Flies were gently aspirated into DAM tubes containing 5% sucrose, 1% agar around Zeitgeber time (ZT) 6, and acclimated overnight. Baseline sleep/activity was determined by averaging three consecutive days of data. Sleep and wake parameters were calculated using a custom Microsoft Excel-based file.

### Nighttime Video Tracking

Flies were gently aspirated into DAM tubes containing 5% sucrose, 1% agar around ZT 6. The tubes were then placed on an infrared light box (140 LED Night Vision Illuminator Lamp) with a light diffuser to observe overnight locomotion in an environmental chamber maintained at 25°C and 65% humidity. A night vision web camera (Agama V-132 1.3M Pixel) was mounted ~15 cm above the flies, and 640X480 resolution still images were taken every 5 sec using Yawcam (free Java software available at yawcam.com). pySolo software [30] was used to analyze locomotion, and nighttime sleep/wake parameters were calculated.

### Drug Treatment

Carbamazepine (CBZ) was obtained from Sigma-Aldrich (St. Louis, MO) and CBZ treatment was adapted from a previously published protocol [31]. CBZ was solubilized in 45% (2-hydroxypropyl)-ß-cyclodextrin (Sigma-Aldrich) to produce a 40 mg/ml stock solution. Following a baseline day, flies were transferred to 5% sucrose, 1% agar medium containing vehicle or CBZ at ZT 8, and nighttime sleep/wake parameters were calculated.

### Circadian Rhythm Analysis

Circadian rhythmicity was analyzed under nearly the same conditions as basal sleep; the only exception was the experimental lighting conditions. Specifically, female flies were subjected to 5 days of 12 hr LD conditions, then 7 days of constant darkness. Free running-period length (tau, **τ**) was calculated using ChronoShop, a software package developed for period analysis [32].

### Sleep Deprivation Experiment

Sleep deprivation experiments were performed using DAM5 monitors in an environmental chamber maintained at 25°C and 65% humidity. To perform sleep deprivation, we used the apparatus and protocol described in [33]. Briefly, the monitors were housed inside a framed box that rotates 180° at a speed of 2–3 revolutions/min once every ~5 min. The monitors dropped approximately 6 cm each rotation, producing a mechanical shock. After one baseline day, 24 hr sleep deprivation commenced at ZT 0. The flies were then given 24 hr to recover. Cumulative sleep loss and rebound were determined relative to each fly’s baseline day. Percent change in sleep for each fly was determined by the formula [(24 hr recovery day sleep – 24 hr baseline day sleep) / 24 hr baseline day sleep] x 100.

### Heat-Induced Seizure Assay

Following a 24 hr of no treatment or sleep deprivation, 3–5 day old flies were individually transferred to empty glass vials (15 mm x 45 mm). After 15–30 min of acclimation, vials were submerged in a 40**°**C waterbath for 2 min. Occurrence or absence of seizing in individual flies was determined every 5 sec, and the proportion of flies seizing at each time point was calculated. Heat-induced seizures were defined as a failure to maintain standing, twitching of the leg, flapping of a wing(s) or curling of the abdomen [29].

### Statistics

All data presented in this study were generated from two or more independent sets of experiments, with the exception of the longevity analysis, which was carried out once. Unless otherwise stated, “n” represents number of total flies examined. Statistical analyses were performed using SigmaPlot 13.0 (Systat Software, Inc., Point Richmond, CA). ANOVA on Rank (Kruskal-Wallis) and Mann-Whitney Rank Sum tests were employed for multiple and pairwise comparisons, respectively. Most data are represented as box plots, with “X” denoting mean values.

## Results

### GEFS+ mutants show increased nighttime sleep and decreased sleep latency

**Fig 1:**
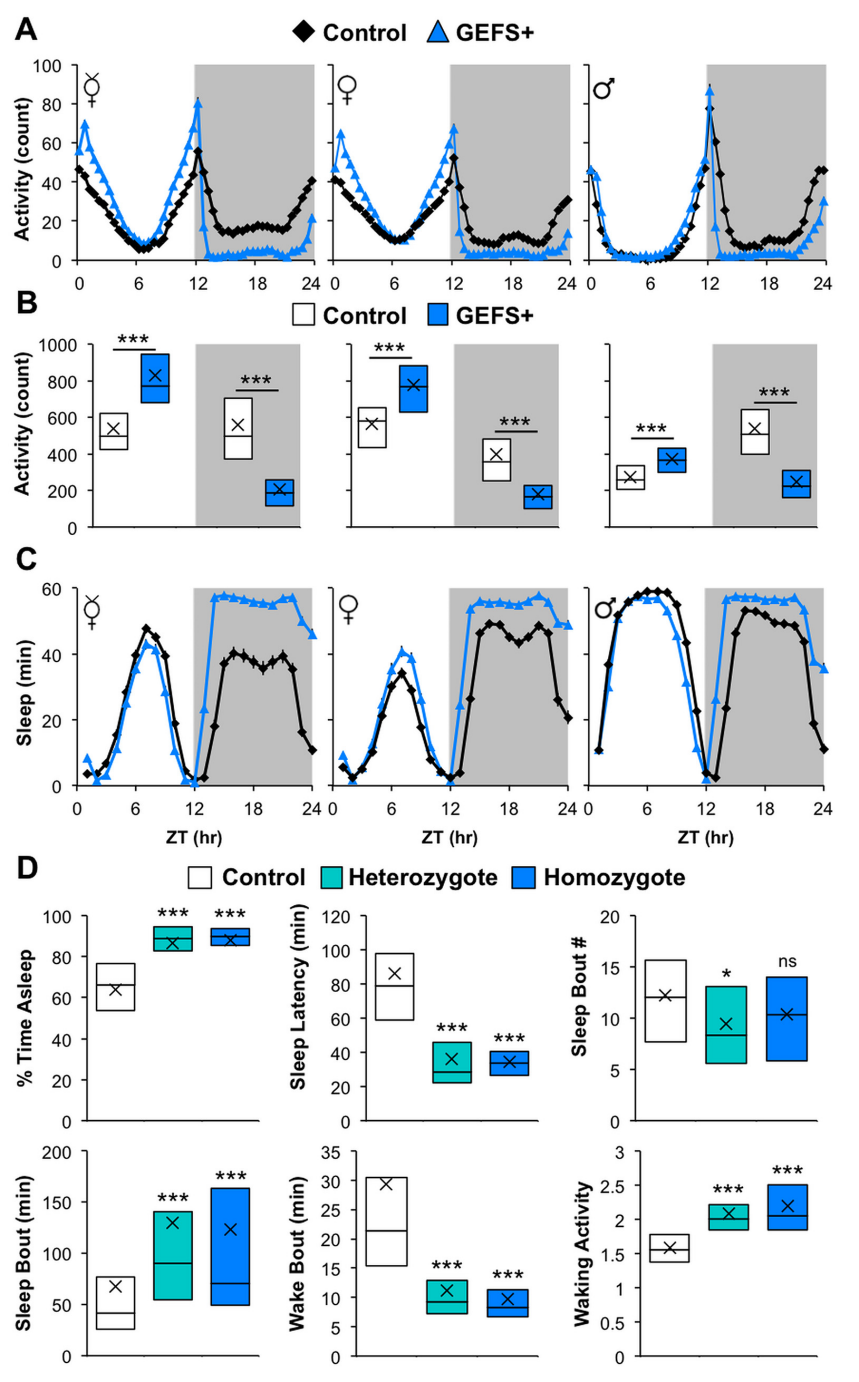
GEFS+ mutation affects sleep/wake behavior. **(A)** The 24 hr activity profiles, **(B)** 12 hr LD activity counts, and **(C)** 24 hr sleep profiles of virgin females (☿), mated females (♀), and males (♂) for knock-in controls (*n* = 85, 93, 95) and GEFS+ mutants (*n* = 88, 87, 94). **(D)** Nighttime 12 hr sleep/activity parameters of mated females for control (*n* = 93), GEFS+ heterozygotes (*n* = 44), and GEFS+ homozygotes (*n* = 87). Data are presented as averages with SEM for **(A, C)** or boxplots with means (“X”) for **(B, D)**. ANOVA on Ranks, Dunn’s compared to control; *p < 0.05, ***p < 0.001.

To examine how an epileptogenic mutation in the *Drosophila* VGSC gene affects fly sleep, we assessed the sleep/wake behavior of the knock-in GEFS+ mutants and their genetic controls generated by Sun *et al*. [29]. Using the *Drosophila* Activity Monitoring (DAM) system, activities of virgin females, mated females and males were monitored for three days at 25**°**C under standard 12 hr LD conditions. Fig 1A shows daily activities of the mutants and control flies averaged over three days. Homozygous GEFS+ virgin and mated females, and hemizygous males exhibited increased daytime activity relative to controls. In contrast, nighttime activity counts were markedly reduced in GEFS+ flies (Fig 1A and 1B). Notably, the activities of GEFS+ mutants sharply dropped upon lights off (Fig 1A).

Despite the greater total daytime activity of flies with the GEFS+ mutation, the effect on daytime sleep was relatively minor (Fig 1C). In contrast, the reduced nighttime activity of GEFS+ mutants coincided with a dramatic increase in sleep amount (Fig 1C). For subsequent analyses of GEFS+ sleep, we focused on mated females and their nighttime sleep unless otherwise noted. To further characterize the effect of the GEFS+ mutation on nighttime sleep, various sleep-related parameters during the scotophase (dark phase) were calculated for homozygous mutants, heterozygous mutants, and control flies (Fig 1D). Corresponding to the rapid decline in activity of GEFS+ mutants after lights off, sleep latencies (time to first sleep episode after lights off) were considerably reduced in the mutants. The exaggerated nighttime sleep of GEFS+ mutant flies was primarily attributed to longer duration of sleep bouts and shorter duration of wake bouts (Fig 1D). Together, these observations indicated that both the onset and maintenance of sleep were promoted by the GEFS+ mutation. Despite spending less time awake at night than controls, GEFS+ mutant flies actually had increased waking activity (counts per waking minute), showing that increased nighttime sleep was not simply caused by a general reduction in locomotor capacity.

We also analyzed sleep in the GEFS+ and control flies at higher resolution using a video tracking system, in which the positions of each fly were assessed every 5 sec. As expected, the video analysis estimated total sleep time at lower values compared to the DAM-based analysis, but recapitulated many of the characteristic features of GEFS+ nighttime sleep, i.e. increases in total sleep time and sleep bout length, and decreases in sleep latency and wake bout length (S1 Fig).

*Drosophila* GEFS+ mutants display a semi-dominant heat-induced seizure phenotype, with significantly less severe seizure events in heterozygous compared to homozygous mutants [29]. In contrast, the effect of the GEFS+ mutation on nighttime sleep is fully dominant, with sleep parameters for GEFS+ heterozygotes and homozygotes being indistinguishable (Fig 1D). Dominantly inherited human *SCN1A* mutations associated with GEFS+ vary with respect to both penetrance and expressivity [24, 34–36]. To determine whether the sleep phenotype of the GEFS+ mutant observed here occurs in other genetic backgrounds, control and GEFS+ homozygous females were out-crossed once to two standard laboratory strains (*Canton-S* and *w* ^*1118*^). Heterozygous female progeny from both crosses also displayed increased nighttime sleep and reduced sleep latency compared to those of genetic controls, albeit with reduced severity (S2 Fig). Thus, although genetic backgrounds significantly influence sleep, the GEFS+ mutant sleep phenotype was detectable in heterozygotes of different genetic backgrounds.

### The GEFS+ mutation has no effect on lifespan

The sleep phenotypes of GEFS+ mutants are the opposite of those of other seizure-prone fly mutants such as *Shaker* (*Sh*), *quiver/sleepless* (*qvr/sss*) and *Hyperkinetic* (*Hk*), all of which display significantly reduced sleep [18–20]. Because these other mutants all have decreased longevity, we examined our GEFS+ model for effects on lifespan. Under normal rearing conditions (12 hr LD cycle at 25°C and 65% humidity), there was no significant effect of GEFS+ mutation on the lifespan of virgin females (S3 Fig).

### Sleep is differentially affected by GABAergic manipulation in GEFS+ mutants and control flies

**Fig 2:**
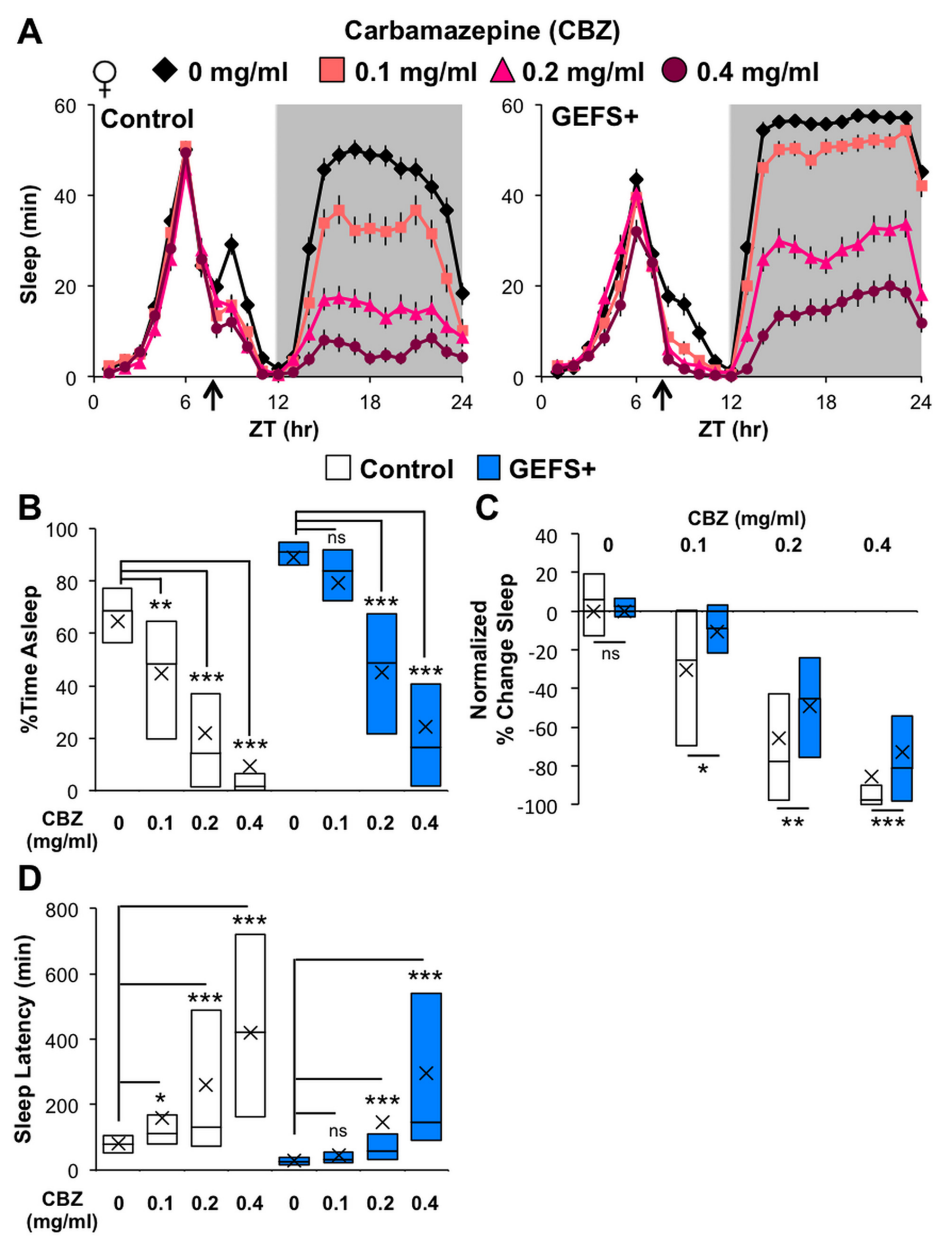
Pharmacologic suppression of GABA_A_ receptor function differentially affects sleep in control and GEFS+ flies. **(A)** Sleep profiles of control (*n* = 63, 60, 62, 60) and GEFS+ (*n* = 62, 61, 63, 62) flies fed vehicle or various concentrations of CBZ starting at ZT 8 (arrow). **(B)** CBZ feeding decreased nighttime sleep; ANOVA on Ranks, Dunn’s within genotype compared to vehicle-fed flies. **(C)** The percent change of nighttime sleep normalized within genotype to vehicle-fed flies revealed that GEFS+ mutants were more resistant to CBZ as compared to control flies at each CBZ concentration; Rank Sum Tests. **(D)** CBZ feeding increased sleep latency; ANOVA on Ranks, Dunn’s within genotype compared to vehicle-fed flies. Data are presented as averages with SEM **(A)** or boxplots with means (“X”) **(B-D)**; *p < 0.05, **p < 0.01, ***p < 0.001.

As observed in mammals, the inhibitory GABAergic system significantly regulates sleep in *Drosophila* [9, 31, 37–39]. Specifically, previous studies have demonstrated that circadian-regulated GABAergic inhibition promotes the initiation and maintenance of sleep [31, 38, 39]. In fact, *Rdl* ^*MDRR*^ mutants, which have enhanced GABAergic transmission due to altered channel properties of the GABA_A_ receptor (Resistant to dieldrin; Rdl), show sleep phenotypes similar to those of GEFS+ mutants ─ shorter sleep latency and increased sleep. Electrophysiological analyses at room temperature (23**°**C) have demonstrated that the *Drosophila* GEFS+ sodium channels display reduced activation thresholds as compared to controls in adult brain GABAergic interneurons, indicating that GABAergic inhibition is increased in the context of the mutant channel [29]. Therefore, we hypothesized that enhanced GABAergic transmission was the primary cause of the GEFS+ sleep phenotypes we observed. To investigate this possibility, we pharmacologically targeted the *Drosophila* Rdl GABA_A_ receptor using carbamazepine (CBZ). In flies, CBZ reduces GABAergic transmission by accelerating the desensitization of Rdl, and it decreases total sleep and increases sleep latency in a dose-dependent manner [31]. As expected, CBZ feeding reduced nighttime sleep and extended sleep latency in a dose-dependent manner in both control and GEFS+ flies (Fig 2A). However, while the lowest concentration of CBZ (0.1 mg/ml) significantly influenced nighttime sleep and sleep latency in control flies, its effects did not reach statistical significance in GEFS+ mutants (Fig 2B and 2D). Furthermore, when the nighttime sleep of CBZ-fed flies was normalized to that of vehicle-fed flies of the same genotype, GEFS+ mutants showed significantly less percent change in nighttime sleep relative to control flies at every CBZ concentration (Fig 2C). Based on these results, GEFS+ mutants appear to be unusually resistant to pharmacologic suppression of GABAergic transmission.

**Fig 3:**
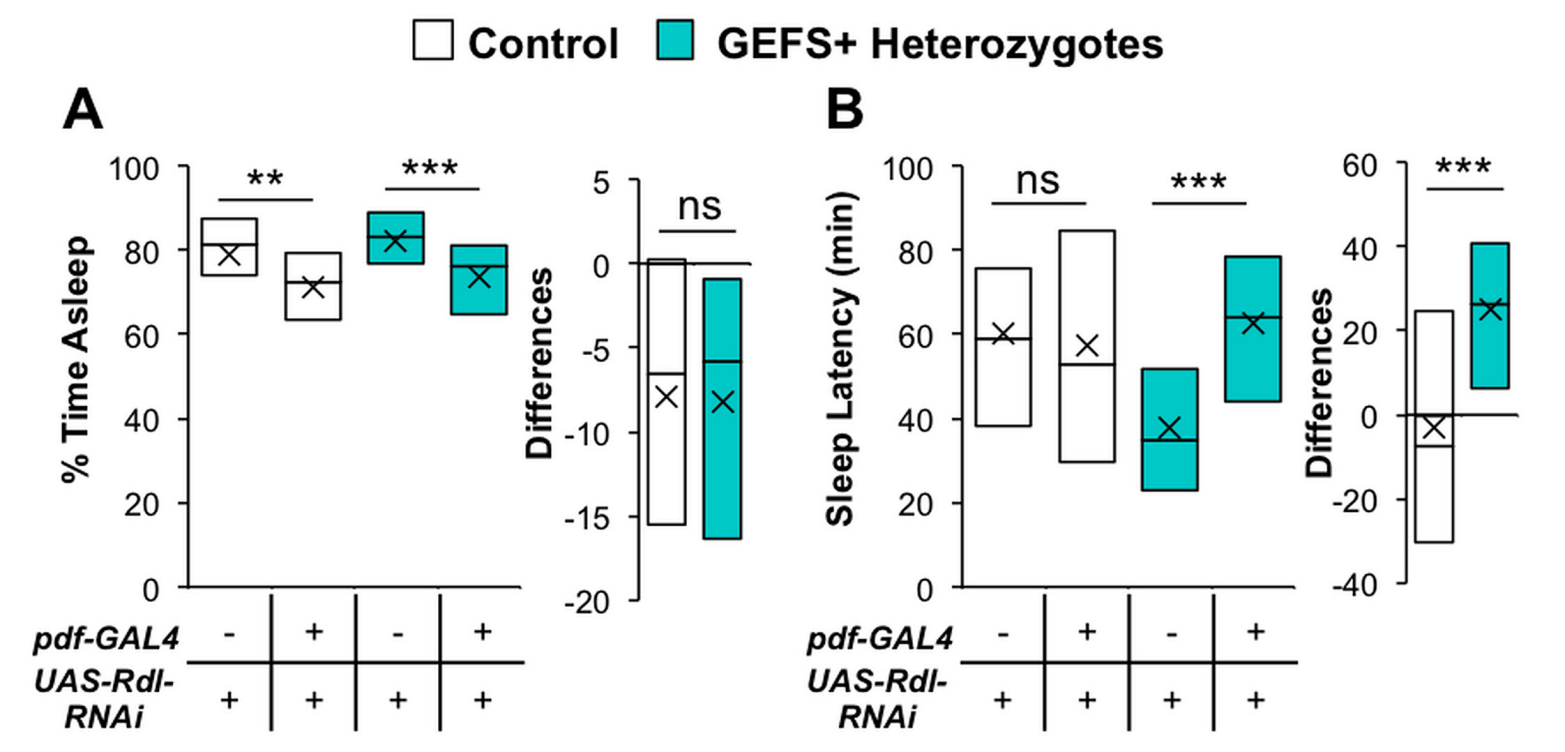
*Rdl* GABA_A_ knockdown in PDF-positive neurons differentially influences sleep latency in GEFS+ mutants. **(A, B)** *Rdl* knockdown in PDF neurons of control and heterozygous GEFS+ mutants; +*/Rdl-RNAi* (*n* = 55), *pdf-GAL4/Rdl-RNAi* (*n* = 49), *GEFS+/+;* +*/Rdl-RNAi* (*n* = 56), *GEFS+/+; pdf-GAL4/Rdl-RNAi* (*n* = 38). **(A)** *Rdl* knockdown in PDF neurons reduced sleep to the same extent in both control and GEFS+ flies. **(B)** *Rdl* knockdown in PDF neurons specifically increased sleep latencies in heterozygous GEFS+ mutants (but not in control flies), restoring the GEFS+ short sleep latencies to control levels; ANOVA on Ranks, Dunn’s Multiple Comparisons. To determine the extent of change caused by *Rdl* knockdown, differences within a genotype were calculated by subtracting experimental data to the averages of RNAi only controls; Rank Sum Test. All data presented as boxplots with means (“X”); **p < 0.01, ***p < 0.001.

GABAergic transmission regulates fly sleep largely through the inhibition of wake-promoting clock neurons that express the neuropeptide pigment dispersing factor (PDF) [38–40]. To examine the role of GABA and PDF neurons in control and mutant flies we knocked down the expression of Rdl GABA_A_ receptors specifically in PDF neurons using *pdf-GAL4* and *UAS-Rdl-RNAi*. Total nighttime sleep was reduced in both control flies and GEFS+ heterozygous mutants upon PDF neuron-specific *Rdl* knockdown (Fig 3A). This result was expected in light of the decreased inhibition of wake-promoting PDF neurons resulting from *Rdl* knockdown, and consistent with the results from previous studies [38, 39]. The extent of nighttime sleep reduction caused by *Rdl* knockdown, which was calculated by finding the difference between experimental data (*pdf-GAL4*/+; *UAS-Rdl-RNAi*/+) and the average of control data (+/+; *UAS-Rdl-RNAi*/+) for each genotype, was not significantly different between control flies and GEFS+ heterozygous mutants (Fig 3A).

Unlike total nighttime sleep, sleep latency of control flies and GEFS+ heterozygous mutants were differentially influenced by *Rdl* knockdown in PDF neurons (Fig 3B). Under the conditions we used, PDF neuron-specific *Rdl* knockdown did not increase sleep latency in control flies, which is inconsistent with a previous observation [38]. This is probably due to the fact that only a single copy of each transgene (*pdf-GAL4* and *UAS-Rdl-RNAi*) was used here. We found, however, that *Rdl* knockdown did significantly increase the sleep latency of GEFS+ heterozygous mutants, restoring sleep latencies in GEFS+ mutants to levels comparable to those in their genetic controls. Further, the extent of change caused by *Rdl* knockdown between the sleep latencies within control flies and GEFS+ mutants was statistically significant (Fig 3B).

### Effects of altered nighttime lighting conditions on GEFS+ sleep phenotypes

**Fig 4:**
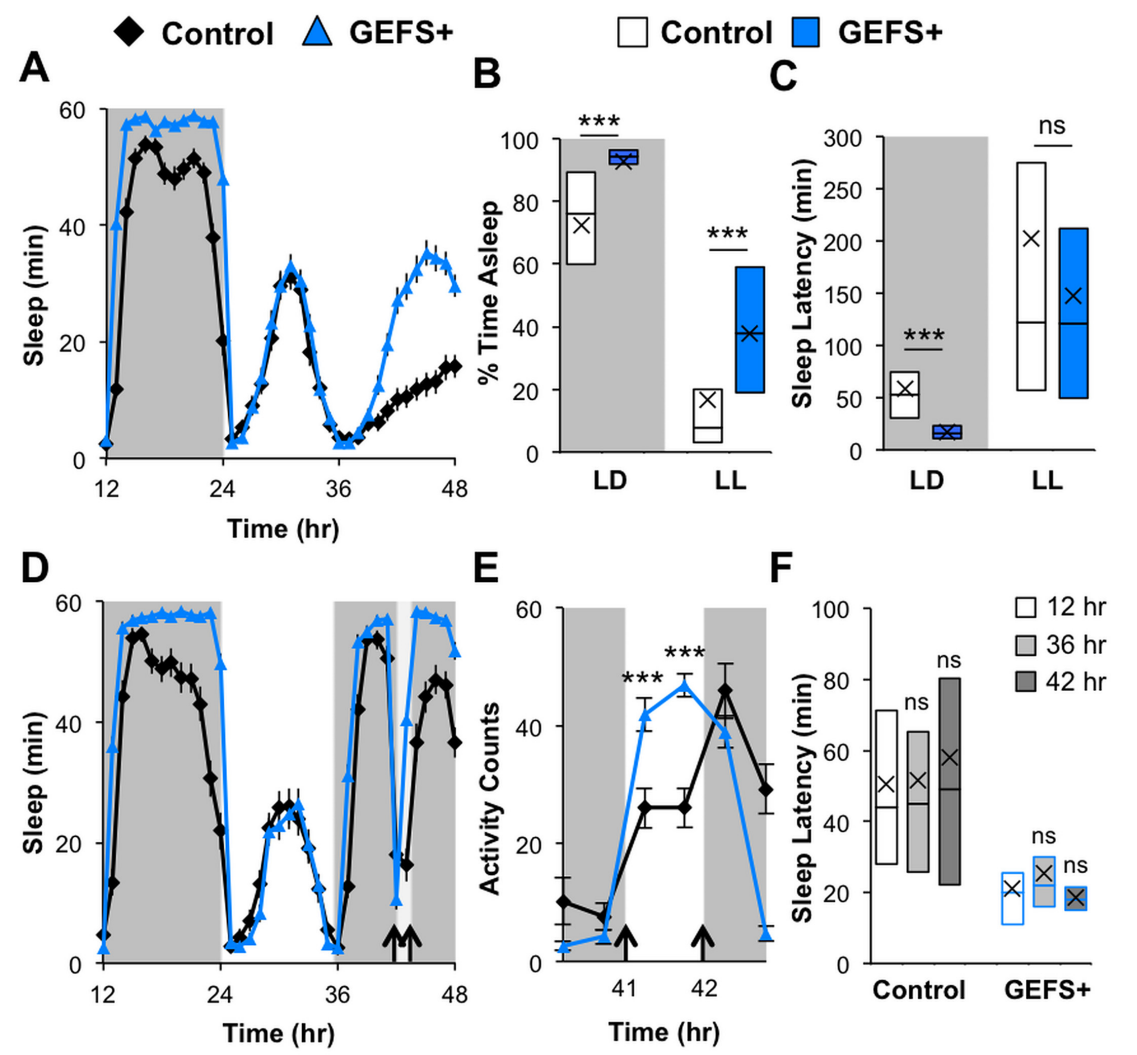
Effect of constant and acute light during the scotophase on GEFS+ and control sleep. **(A)** Sleep profiles of control (*n* = 83) and GEFS+ (*n* = 95) flies under constant light/light (LL) exposure. **(B)** Total sleep and **(C)** sleep latency during subjective night under LD and LL conditions; Rank Sum Tests. **(D)** Sleep and **(E)** activity profiles of control (*n* = 52) and GEFS+ (*n* = 59) flies subjected to a 1 hr scotophase light pulse; repeated measures ANOVA on Ranks. **(F)** Sleep latencies of control and GEFS+ flies after normal lights off (12 hr and 36 hr) and the scotophase pulse (42 hr); ANOVA on Ranks, Dunn’s vs 12 hr control. Data presented as averages with SEM (**A**, **D** and **E**) or boxplots with means (“X”) (**B**, **C** and **F**); ***p < 0.001.

GEFS+ mutants showed phase-specific abnormalities in locomotor activity ─ an increase during photophase and a decrease in scotophase as compared to genetic controls. Their sleep abnormalities were primarily observed during the scotophase (Fig 1). Furthermore, the effect of the GEFS+ mutation on short sleep latency depended on the level of *Rdl* in light-activatable PDF-positive neurons (Fig 3B). These observations indicate that light may play an important role in the expressivity of the GEFS+ sleep phenotype. To better understand how light influences GEFS+ sleep, sleep/wake activity was analyzed under abnormal nighttime lighting conditions. When exposed to constant light following a baseline light/dark day, both control flies and GEFS+ mutants dramatically reduced subjective nighttime sleep (Fig 4A and 4B). Under this constant light condition, GEFS+ mutants still spent considerably more time asleep than controls. Sleep latencies at subjective dusk (36 hr from start of baseline day) of both control and GEFS+ flies were lengthened in response to constant lighting conditions, eliminating the short sleep latency phenotype of GEFS+ mutants (Fig 4C).

Nocturnal light interruption has been shown to promptly induce waking [41], so we examined behavioral responses of control and mutant flies to light during the scotophase. Following a baseline light/dark day, flies were subjected to a 1 hr light stimulus five hours after lights off (41–42 hr from start of baseline day). Both control and GEFS+ flies robustly suppressed sleep in response to the acute light exposure (Fig 4D). Interestingly, GEFS+ mutants were significantly more active during the light stimulus relative to controls as judged by the total number of activity counts (Fig 4E). Upon light removal (42 hr), sleep latencies in control and mutant flies were consistent with those seen at normal lights off times (12 hr and 36 hr), and, therefore, the effect of GEFS+ mutation on rapid sleep onset was still observed (Fig 4F). These results show that GEFS+ mutants can be aroused from their exaggerated nighttime sleep by light stimuli, become hyperactive during scotophase light, and maintain the propensity to rapidly initiate sleep in response to a lights off signal during the night.

### GEFS+ mutants have normal circadian locomotor rhythms

**Fig 5:**
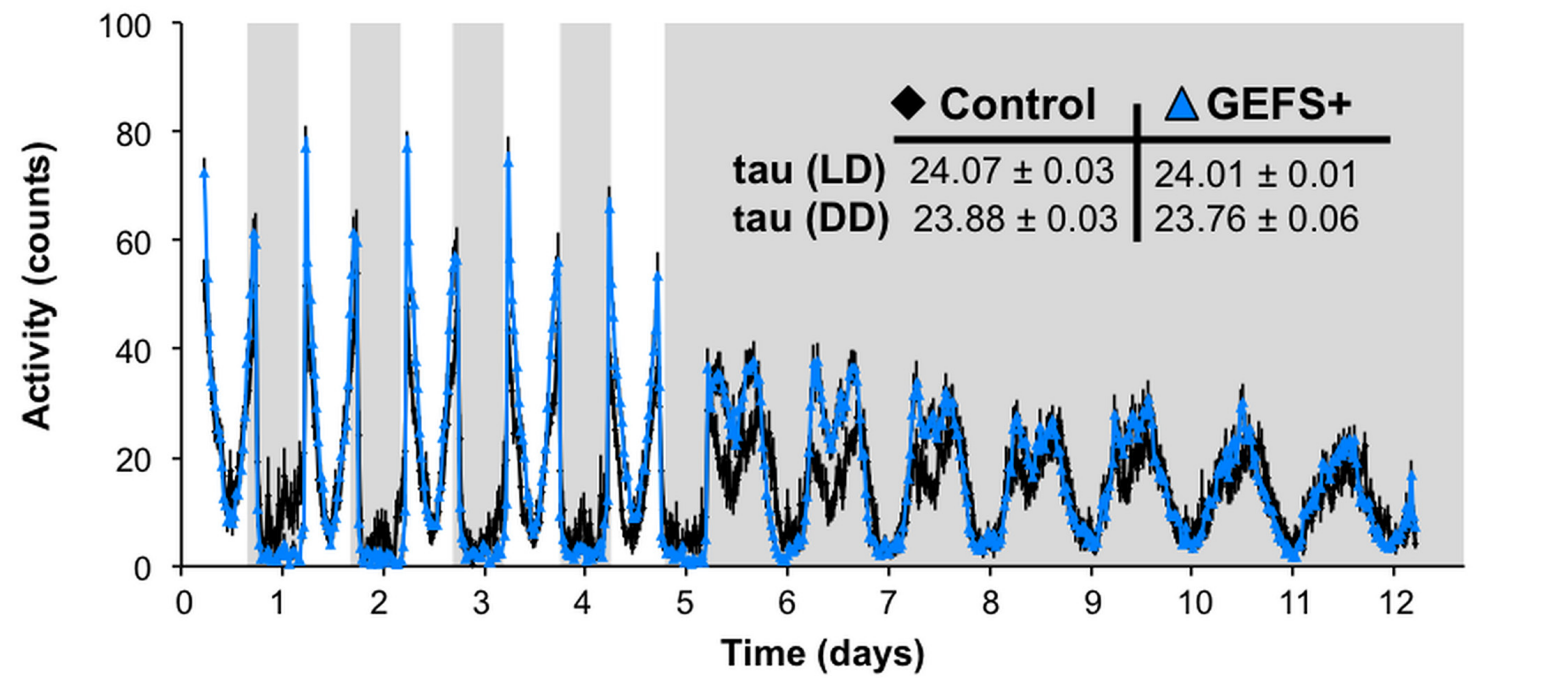
Circadian regulation is intact in GEFS+ mutants. Locomotor activity profiles of control (*n* = 22) and GEFS+ (*n* = 30) flies during 5 days in light/dark (LD) followed by then 7 days in constant dark (DD). GEFS+ flies show normal circadian rhythmicity under both LD and DD treatment (inset). Data presented as averages with SEM.

Fly sleep is regulated by circadian and homeostatic mechanisms. To explore whether the GEFS+ mutation alters circadian regulation, free-running activity rhythms of control flies and GEFS+ mutants were examined during seven days of constant darkness following five days of 12 hr LD. As determined by χ^2^ periodogram analyses, both GEFS+ and control flies displayed robust rhythmicity in dark/dark conditions and maintained comparable mean circadian periods (Fig 5), indicating that the GEFS+ mutation does not have a significant effect on the central circadian clock.

### GEFS+ mutants are defective in homeostatic regulation of sleep

**Fig 6:**
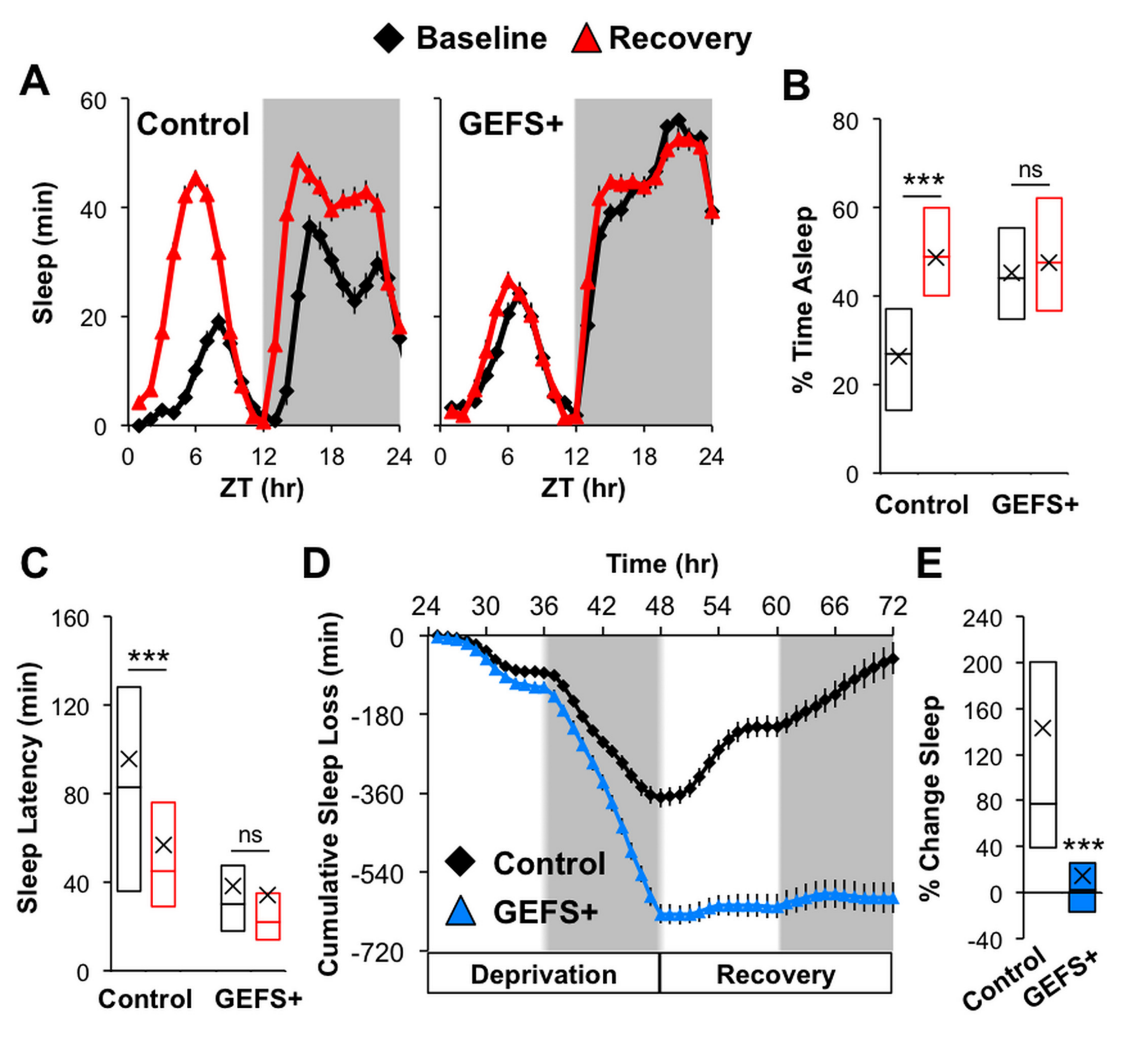
GEFS+ mutants lack homeostatic sleep regulation. **(A)** The 24 hr sleep profiles of baseline day and recovery day following 24 hr sleep deprivation in control (*n* = 81) and GEFS+ mutants (*n* = 86). **(B)** The percentage of time asleep over the 24 hr period and **(C)** subjective sleep latencies for baseline and recovery days; ANOVA on Ranks, Dunn’s compared to baseline data within genotype. **(D)** Cumulative sleep loss during 24 hr sleep deprivation and recovery. Sleep debt is presented relative to baseline sleep for each genotype. **(E)** Percent change in 24 hr sleep compared between before and after sleep deprivation; Rank Sum Test. Data presented as averages with SEM **(A, D)** or boxplot with means (“X”) **(B, C, E)**; ***p < 0.001.

Next, we examined whether the GEFS+ mutation affects homeostatic sleep regulation. Following a baseline day, flies were mechanically deprived of sleep for 24 hr using the protocol described in [33] and then given one day to recover. Control flies exhibited shorter sleep latency following sleep deprivation and recovered all of their sleep loss by the end of the recovery period (Fig 6). In striking contrast, GEFS+ mutants had a severe impairment in homeostatic regulation, and showed almost no sleep rebound after 24 hr of deprivation. The defects in homeostatic regulation of sleep were also observed, at similar levels, in heterozygous GEFS+ mutants (data not shown).

### Sleep deprivation affects seizure susceptibility

**Fig 7:**
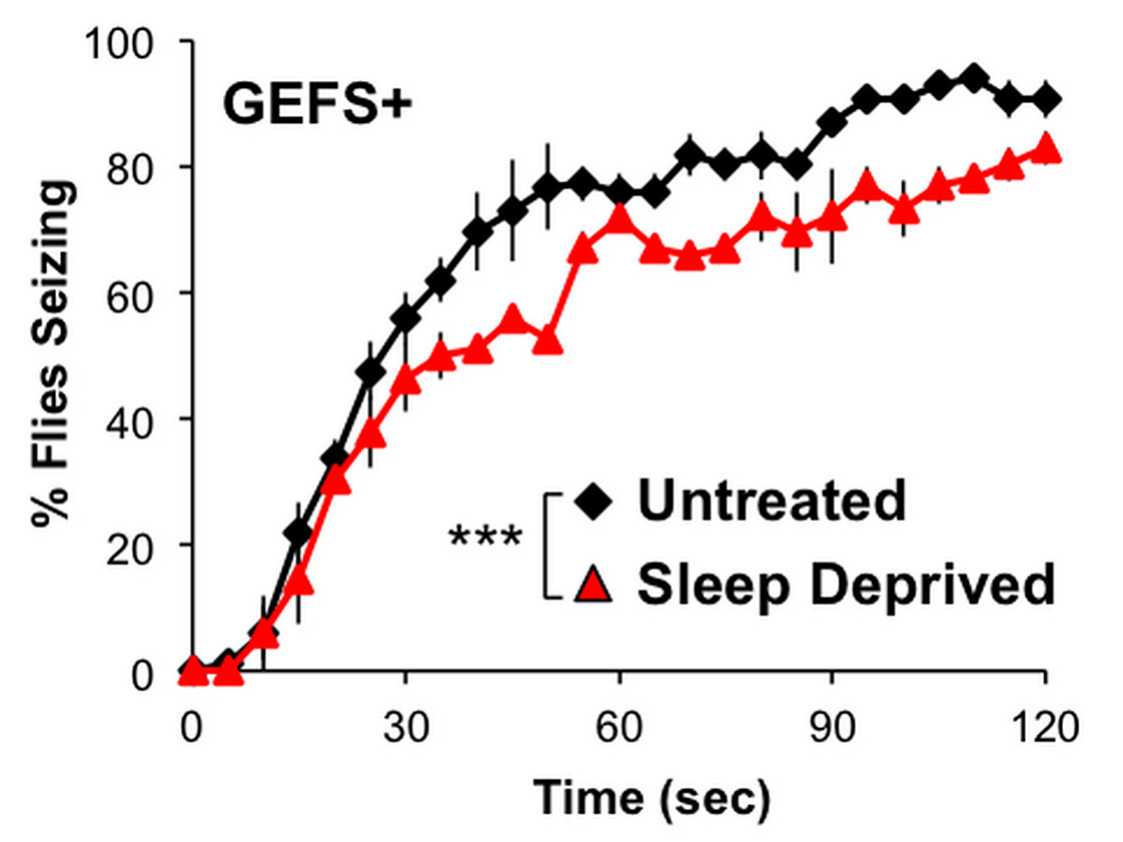
Sleep deprivation reduces heat-induced seizure susceptibility of the GEFS+ mutant. Percentage of seizing flies for untreated and 24 hr sleep-deprived GEFS+ mutants when exposed to 40°C; two-way repeated measures ANOVA, Holm-Sidak Multiple Comparisons. Data presented as averages of three independent experiments (*n* = 16, 28, 25 for untreated and *n* = 14, 27, 27 for deprived) with SEM; ***p < 0.001.

Since sleep deprivation is a common trigger of seizure in epileptic patients [4, 5], we investigated whether 24 hr sleep deprivation affects the heat-induced seizure susceptibility of control and GEFS+ flies. Seizure susceptibility between ZT 0 and ZT 1 was assessed using the protocol described in Sun *et al*. (2012). Without sleep deprivation, control flies did not show any seizure-like behavior during a 2 min exposure to 40°C (0%, *n* = 57). However, a significant proportion of control flies had at least one heat-induced seizing episode after sleep deprivation (14%, *n* = 63; *p* < 0.01 Fisher’s exact test). In contrast, the severity of the GEFS+ seizure phenotype was reduced after being sleep deprived. Sleep-deprived GEFS+ mutants were less likely than untreated GEFS+ mutant counterparts to have a seizure during a 2 min 40**°**C exposure (Fig 7). Also, while all untreated GEFS+ mutants displayed at least one seizure, a portion of the sleep-deprived mutants never had a seizure during the observation period (100%, *n* = 69 vs 94%, *n* = 68).

## Discussion

To investigate the effects of an epilepsy-causing VGSC mutation on sleep, we characterized the sleep/wake behavior of mutant flies harboring a knock-in *SCN1A* mutation (K1270T) that confers human GEFS+. We found that *Drosophila* GEFS+ mutants displayed distinct abnormalities in sleep regulation; compared to appropriate genetic controls, GEFS+ mutants fell asleep significantly faster at lights off and slept significantly longer at night (Fig 1). The sleep phenotype of GEFS+ flies was robust enough to be observed regardless of sex, mating state or genetic background, and was strongly penetrant in heterozygotes (Fig 1, S1 and S2 Figs).

Although the tested *Drosophila* GEFS+ mutant has seizures at high temperatures, its sleep phenotype is the opposite of those of other seizure-prone, hyperexcitable fly mutants such as *Sh*, *Hk* and *qvr*/*sss*, all of which have reduced sleep [18–20]. The unique GEFS+ sleep phenotype most likely reflects the gain-of-function properties of the mutant VGSCs [29]. Previous electrophysiological analyses of *Drosophila* GABAergic interneurons in the brain indicated that at room temperature (23°C) the GEFS+ mutation leads to hyperpolarization of the threshold voltage for activating sodium currents, resulting in increased GABAergic inhibitory activity as compared to controls [29]. At high temperature (35°C), this GEFS+ mutation has a deleterious gain-of-function effect in GABAergic interneurons, significantly increasing sodium currents due to defective channel inactivation and prolonging post-stimulus depolarization, which reduces GABAergic activity and induces seizures [29]. Our findings in this study support involvement of the GABAergic inhibitory system in the manifestation of GEFS+ sleep phenotypes. As shown in Fig 2, GEFS+ mutants were more resistant than control flies to the sleep-suppressing effect of carbamazepine (CBZ), a drug that accelerates desensitization of the *Drosophila* GABA_A_ receptor Rdl. Although we did not rule out the possibility that GEFS+ mutants ate less CBZ-containing food than control flies, our results, together with the previous electrophysiological findings, suggest that GABAergic transmission is indeed enhanced in GEFS+ mutants. Resistance to CBZ has also been observed in gain-of-function *Rdl* ^*A302S*^ mutants, which have increased total sleep and short sleep latencies [31] ─ sleep phenotypes similar to those of GEFS+ mutants.

The wake-promoting PDF-positive neurons are essential targets of GABAergic inhibition in *Drosophila* sleep regulation [38, 39, 42–44]. We found that total sleep time decreased and sleep latencies increased in GEFS+ mutants when *Rdl* expression was knocked down using RNAi specifically in PDF-positive neurons (Fig 3). Interestingly though, under the conditions we used, the sleep latencies of control flies were not significantly affected by this genetic manipulation, indicating the increased sensitivity of sleep latencies to knockdown of *Rdl* expression in GEFS+ mutants as compared to control flies. These findings seem contradictory to our results with CBZ feeding, where GEFS+ mutants were more resistant than controls to functional suppression of Rdl. However, we think that these data are consistent with the idea that enhanced inhibitory GABAergic transmission to PDF-positive neurons underlies GEFS+ sleep phenotypes. Our interpretation is that PDF neurons express extra Rdl channels, which, although not necessary for normal GABAergic transmission, are required to respond to increased GABA release from GABAergic neurons. Thus, CBZ must block extra Rdl channels in GEFS+ mutants to compete with their increased GABAergic transmission. Further, mild genetic knockdown of *Rdl* (one copy of *pdf*-*GAL4* and *UAS-Rdl-RNAi* transgenes) removes the extra Rdl channels, preferentially affecting enhanced GABAergic transmission in GEFS+ mutants, but having less effect on normal GABAergic transmission in control flies. This would explain why sleep is more resistant to CBZ feeding, yet sleep latencies are more sensitive to *Rdl* knockdown in PDF neurons, in GEFS+ compared to control flies.

A characteristic feature of GEFS+ mutants is their photophase- and scotophase-specific abnormalities in locomotor activity and sleep (Fig 1). Namely, locomotor activity of GEFS+ mutants is increased during the photophase, but decreased during the scotophase, when compared to control flies (Fig 1). In addition, GEFS+ mutation preferentially affects nighttime sleep (Fig 1C and 1D). While GEFS+ mutant VGSCs could affect the neuronal outputs of endogenous rhythm behaviors, our experiments under different light conditions suggest that the light stimulus is a major factor responsible for the phase-specific phenotypes of GEFS+ mutants (Fig 4). We found that a lights off signal was required to observe the rapid sleep onset of GEFS+ mutants at dusk. In addition, turning on the light in the middle of subjective nighttime resulted in hyperactive GEFS+ mutants (Fig 4E), and removal of the light caused rapid sleep initiation similar to sleep onset at normal dusk. Together these findings indicate that the GEFS+ mutation may increase the sensitivity of relevant neuronal circuits and narrow the buffering of behavioral responses to light on and off stimuli.

Considering that dysregulation of PDF neurons likely causes the abnormal sleep in GEFS+ mutants, it was interesting to find that mutants did not display rhythmicity problems during free-running dark/dark conditions (Fig 5A). Among PDF-expressing cells, the small ventrolateral neurons (sLNvs) are considered the main pacemakers for circadian activity [45, 46], while large ventral lateral neurons (lLNvs) distinctly mediate light-dependent arousal [40]. Our results indicate that VGSC modification by the GEFS+ mutation does not affect the circadian sLNvs function, but significantly influences lLNv activity.

In addition to basal sleep abnormalities, the GEFS+ mutants showed a severe defect in homeostatic sleep rebound after 24 hr sleep deprivation (Fig 6). Although the neural mechanisms underlying sleep homeostasis remain unclear, it is thought that the central nervous system monitors and evaluates the quality and quantity of sleep, and then facilitates compensatory sleep by altering the sleep rheostat. A recent study demonstrated that chronic sleep deprivation enhances the excitability of PDF-positive lLNvs [47]. This finding is counterintuitive since lLNvs are wake-, but not sleep-, promoting neurons. Perhaps the lLNv excitability induced by sleep deprivation serves as a signal of sleep loss, which subsequently results in the activation of sleep-promoting circuits. It is possible that GEFS+ mutant is defective for such a signal because of enhanced GABAergic inhibition on lLNvs. Alternatively, the GEFS+ mutation may affect the output arm of the sleep homeostat. A recent study has shown that sleep deprivation increases the neuronal excitability of sleep-promoting neurons that project to the dorsal fan-shaped body (FB) [48]. Therefore, the GEFS+ mutation may directly or indirectly enhance inhibition on sleep-promoting dorsal FB neurons, thereby reducing deprivation-induced excitability and preventing sleep restoration. Future studies are needed to determine how altered sodium channel function in GEFS+ mutants results in the disruption of homeostatic sleep regulation.

Sleep deprivation often exacerbates the onset, frequency and severity of seizures in epileptic patients and rodent models of epilepsy. We found that control flies showed a greater susceptibility to heat-induced seizures after experiencing 24 hr sleep deprivation. Unexpectedly, the same sleep deprivation reduced the probability of heat-induced seizures in GEFS+ mutants (Fig 7). This result is contrary to a new study showing that *sesB* and *sei* ^*ts1*^mutants increase their bang-sensitivity (vortex and recover assay) following 12 hr of sleep deprivation (using a SNAP device [15]) [21]. Although the mechanism by which sleep deprivation affects seizures is not known, it is hypothesized that sleep deprivation can modify properties of particular neural circuitry involved in seizure susceptibility. For example, the PDF-positive lLNvs have broad dendritic and axonal arbors and exhibit increased excitability following deprivation [47], which may influence the spread of seizure activity. Considering GEFS+ sleep phenotypes, the effect of sleep deprivation on PDF neurons and other neural circuits could be different in GEFS+ mutants, leading to unique alterations in the seizure susceptibility of GEFS+ mutants.

Mouse models of GEFS+ or Dravet mutations in *Scn1a* generally display reduced activity in inhibitory GABAergic neurons, but not in glutamatergic excitatory neurons. This is thought to be due to isoform specific or preferential expression of *Scn1a* in inhibitory neurons [24]. Unlike mammals, which have multiple VGSC genes, *Drosophila* has only one (*paralytic*). The GEFS+ mutation described here is in a constitutively included exon, and the protein produced is expressed throughout the fly. In spite of this, Sun *et al*. inferred stronger effects of GEFS+ mutation on inhibitory GABAergic neurons than on excitatory cholinergic neurons [29]. Our study also shows the critical role of GABAergic inhibition in manifestation of the GEFS+ sleep phenotype. These findings suggest that neurons controlling sleep and seizures exhibit diverse sensitivity to altered neuronal excitability due to VGSC mutation. We propose that further studies using VGSC mutants will help elucidate how sleep is regulated through interactions of multiple neural circuits with distinct electrophysiological properties.

Many human studies, as well as research on murine models of epilepsy, have reported various sleep deficits including abnormal NREM/REM, disrupted circadian rhythm and poor homeostatic rebound [25, 28]. For example, Papale *et al*. reported that heterozygous mouse *Scn1a* mutants carrying a hypomorphic loss-of-function GEFS+ mutation (R1848H) exhibit increased wakefulness and reduced amounts of NREM and REM sleep during the scotophase, but show normal sleep recovery after sleep deprivation [25]. More recently, Kalume *et al*. documented the profound disruption of sleep during the photophase and impaired homeostatic sleep rebound in mice heterozygous for a *Scn1a* null allele, a model of Dravet syndrome (DS) ─ a severe, childhood-onset and often refractory epilepsy syndrome [28]. These studies show that epilepsy-related VGSC mutants could display significantly different sleep phenotypes depending on the severity of reduction in sodium channel function. Although GEFS+ VGSC mutations in both fly and mouse are shown to induce febrile seizures and modulate sleep preferentially through GABAergic inhibitory neurons, there are a number of neuroanatomical and physiological (e.g., body temperature) differences that could account for the differences in their respective sleep phenotypes. Nonetheless, our study using a fly GEFS+ model provides a unique opportunity to further investigate the basic biological mechanisms underlying the effect of an epileptogenic mutation on sleep regulation, as well as on the bidirectional relationships between sleep and seizures.

## Supporting Information

S1 Fig Sleep abnormalities in the GEFS+ mutant scrutinized using video tracking. **A)** Nighttime activity and sleep profiles and **B)** 12 hr sleep parameters of control (*n* = 28) and GEFS+ (*n* = 29) flies in DAM vials determined using pySolo tracking software; Rank Sum Test. Data presented as averages with SEM or boxplots with means (“X”); **p < 0.01, ***p < 0.001.
(DOCX)

S2 Fig The GEFS+ mutation affects sleep regardless of genetic background. Nighttime sleep parameters of control and heterozygous GEFS+ mated females from an outcross with wild-type genetic backgrounds (*CS* and *w* ^*1118*^); *CS* control and GEFS+/+ (*n* = 32, 64), *w* ^*1118*^ control and GEFS+ (*n* = 30, 64); ANOVA on Ranks, Dunn’s Multiple Comparisons. Data presented as boxplots with mean (“X”); **p < 0.01, ***p < 0.001.
(DOCX)

S3 Fig The GEFS+ mutation does not have effect on longevity. Percent survival of control and GEFS+ virgin females. Flies were raised in ~20 flies/vial, at 25°C 65% humidity, and transferred to new vials every 3–4 days; control (*n* = 141), GEFS+ (*n* = 95); Survival Log Rank analysis. Data presented as daily averages of surviving flies in each vial with SEM.
(DOCX)
