# Supplementary material for: Exaggerated Nighttime Sleep and Defective Sleep Homeostasis in a Drosophila Knock-In Model of Human Epilepsy
Source: PLoS One. 2015 Sep 11;10(9):e0137758. doi: 10.1371/journal.pone.0137758 (PMC4567262; doi:10.1371/journal.pone.0137758)
Supplement: S1 Fig — A) Nighttime activity and sleep profiles and B) 12 hr sleep parameters of control (n = 28) and GEFS+ (n = 29) flies in DAM vials determined using pySolo tracking software; Rank Sum Test. Data presented as averages with SEM or boxplots with means (“X”); **p < 0.01, ***p < 0.001. (DOCX) [file pone.0137758.s001.docx]

**S1 Fig. Sleep abnormalities in the GEFS+ mutant scrutinized using video tracking. A)** Nighttime activity and sleep profiles and **B)** 12 hr sleep parameters of control (*n* = 28) and GEFS+ (*n* = 29) flies in DAM vials determined using pySolo tracking software; Rank Sum Test. Data presented as averages with SEM or boxplots with means (“X”); **p < 0.01, ***p < 0.001.
