# Supplementary material for: Exaggerated Nighttime Sleep and Defective Sleep Homeostasis in a Drosophila Knock-In Model of Human Epilepsy
Source: PLoS One. 2015 Sep 11;10(9):e0137758. doi: 10.1371/journal.pone.0137758 (PMC4567262; doi:10.1371/journal.pone.0137758)
Supplement: S2 Fig — Nighttime sleep parameters of control and heterozygous GEFS+ mated females from an outcross with wild-type genetic backgrounds (CS and w 1118); CS control and GEFS+/+ (n = 32, 64), w 1118 control and GEFS+ (n = 30, 64); ANOVA on Ranks, Dunn’s Multiple Comparisons. Data presented as boxplots with mean (“X”); **p < 0.01, ***p < 0.001. (DOCX) [file pone.0137758.s002.docx]

**S2 Fig. The GEFS+ mutation affects sleep regardless of genetic background.** Nighttime sleep parameters of control and heterozygous GEFS+ mated females from an outcross with wild-type genetic backgrounds (*CS* and *w^1118^*); *CS* control and GEFS+/+ (*n* = 32, 64), *w^1118^* control and GEFS+ (*n* = 30, 64); ANOVA on Ranks, Dunn’s Multiple Comparisons. Data presented as boxplots with mean (“X”); **p < 0.01, ***p < 0.001.
