# Supplementary material for: Exaggerated Nighttime Sleep and Defective Sleep Homeostasis in a Drosophila Knock-In Model of Human Epilepsy
Source: PLoS One. 2015 Sep 11;10(9):e0137758. doi: 10.1371/journal.pone.0137758 (PMC4567262; doi:10.1371/journal.pone.0137758)
Supplement: S3 Fig — Percent survival of control and GEFS+ virgin females. Flies were raised in ~20 flies/vial, at 25°C 65% humidity, and transferred to new vials every 3–4 days; control (n = 141), GEFS+ (n = 95); Survival Log Rank analysis. Data presented as daily averages of surviving flies in each vial with SEM. (DOCX) [file pone.0137758.s003.docx]

**S3 Fig. The GEFS+ mutation does not have effect on longevity.** Percent survival of control and GEFS+ virgin females. Flies were raised in ~20 flies/vial, at 25°C 65% humidity, and transferred to new vials every 3-4 days; control (*n* = 141), GEFS+ (*n* = 95); Survival Log Rank analysis. Data presented as daily averages of surviving flies in each vial with SEM.
